# Next-generation sequencing identifies CDKN2A alterations as prognostic biomarkers in recurrent or metastatic head and neck squamous cell carcinoma predominantly receiving immune checkpoint inhibitors

**DOI:** 10.3389/fonc.2023.1276009

**Published:** 2023-10-23

**Authors:** Liqiong Xue, Wenbo Tang, Jiuli Zhou, Junli Xue, Qun Li, Xiaoxiao Ge, Fengjuan Lin, Wei Zhao, Ye Guo

**Affiliations:** Department of Oncology, Shanghai East Hospital, School of Medicine, Tongji University, Shanghai, China

**Keywords:** next-generation sequencing, head and neck squamous cell carcinoma, cyclin-dependent kinase inhibitor 2A, prognosis, immunotherapy

## Abstract

**Background:**

This study aimed to identify potential biomarkers in patients with recurrent or metastatic head and neck squamous cell carcinoma (HNSCC) and further probe the prognostic implications of CDKN2A mutations, particularly within a subset receiving immunotherapy.

**Methods:**

In this retrospective single-center study, we evaluated the next-generation sequencing (NGS) data from Foundation Medicine (FM) for patients with recurrent or metastatic HNSCC between January 1, 2019, and December 31, 2021. Patients were stratified based on CDKN2A loss-of-function (LOF) versus wild-type (WT) categorizations, with a focused subgroup analysis on those administered immunotherapy.

**Results:**

The study encompassed 77 patients, of which 62 had undergone immunotherapy. The median duration of follow-up was 22.6 months. For the CDKN2A LOF group, the median overall survival (OS) was 16.5 months, contrasted with 30.0 months in the CDKN2A WT group (*P*=0.014). Notably, female gender (hazard ratio [HR]=4.526, 95% confidence interval [CI]: 1.934-10.180, *P*=0.0003) and CDKN2A LOF (HR=2.311, 95% CI: 1.156-4.748, *P*=0.019) emerged as independent risk factors for mortality in patients with recurrent or metastatic HNSCC. Within the immunotherapy subset, the median OS was 11.7 months for the CDKN2A LOF group, and 22.5 months for the CDKN2A WT group (*P*=0.017). Further, the female gender (HR=4.022, 95% CI: 1.417-10.710, *P*=0.006), CDKN2A LOF (HR=4.389, 95% CI: 1.782-11.460, *P*=0.002), and a combined positive score below 1 (HR=17.20, 95% CI: 4.134-79.550, *P*<0.0001) were identified as significant predictors of mortality among patients with recurrent or metastatic HNSCC receiving immunotherapy.

**Conclusion:**

Alterations manifesting as LOF in the CDKN2A gene stand as robust indicators of unfavorable survival outcomes in HNSCC patients, including the subset that underwent immunotherapy.

## Introduction

Cancers of the head and neck encompass malignancies in the oral cavity, lip, various segments of the pharynx (including the nasopharynx, oropharynx, and hypopharynx), different regions of the larynx (glottic and supraglottic), ethmoid sinus, maxillary sinus, salivary glands, mucosal melanoma, as well as occult primary tumors within the head and neck region (1–4). In 2020, lip and oral cavity cancers were diagnosed in approximately 377,713 individuals globally, leading to 177,757 fatalities. Simultaneously, pharyngeal cancers accounted for 316,020 new diagnoses and resulted in 166,750 deaths (5). Notably, squamous cell carcinoma histology is observed in about 90% of head and neck cancers, termed head and neck squamous cell carcinoma (HNSCC) (2, 3, 6). Incidence rates are higher among men compared to women (1–3, 6). Prominent risk factors encompass tobacco consumption, excessive alcohol intake, betel quid chewing, familial predisposition, and infections with specific viruses such as the human papillomavirus (HPV) and Epstein-Barr virus (EBV) (1–3, 6). The prognosis remains dismal for patients with recurrent or metastatic HNSCC (1, 3, 6, 7).

Identifying biomarkers indicative of poor prognosis is pivotal in pinpointing HNSCC patients at an augmented risk of unfavorable outcomes. A heightened neutrophil-to-lymphocyte ratio, elevated platelet count, and an increased platelet-to-lymphocyte ratio have been correlated with increased mortality (8–10). Additionally, smoking habits, coupled with the Head and Neck Surgery Risk Index, have associations with mortality (11–13). However, these biomarkers present limitations, primarily because they aren’t rooted in the molecular biology governing the tumor cells’ biological behavior. As such, next-generation sequencing (NGS) emerges as an instrumental technique for expansive screening of genetic alterations within tumors. NGS is advocated for the management of recurrent or persistent HNSCC, facilitating personalized therapeutic strategies based on actionable mutations (6). Nevertheless, NGS can also uncover several non-actionable mutations, offering insights into tumor biology and paving the way for potential drug target identification. Notably, the commercial panel from Foundation Medicine (FM) evaluates the mutational profile of 324 genes frequently altered in cancerous conditions (14).

Cyclin-dependent kinase inhibitor 2A (CDKN2A), a key member of the INK4 family of tumor suppressor genes, plays an intricate role in tumorigenesis, affecting both cell proliferation and angiogenesis (15). In HNSCC, alterations in CDKN2A, such as copy number deletions or reduced p16INK4a expression, correlate with poor prognosis (16). Furthermore, CDKN2A methylation is tied to HNSCC’s onset, progression, and metastasis, reinforcing its diagnostic value (17). Notably, CDKN2A also influences the tumor microenvironment. A recent study highlighted its association with tumor-infiltrating immune cells: elevated CDKN2A expression is linked to increased infiltration by activated B cells and CD4+ T cells, indicating a better prognosis, while its reduced expression is associated with greater neutrophil infiltration, suggesting a worse outcome (18). This underscores CDKN2A’s role in shaping the immune landscape within tumors and its potential impact on prognosis.

Immunotherapy represents a groundbreaking therapeutic approach in HNSCC, enhancing survival outcomes for specific patient cohorts (6, 7). This study sought to elucidate the prognostic implications of CDKN2A mutations in patients with recurrent or metastatic HNSCC, complemented by a nuanced subgroup analysis focusing on those undergoing immunotherapy. Such insights hold potential to refine the therapeutic strategies for patients contending with recurrent or metastatic HNSCC.

## Methods

### Study design and patients

In this single-center retrospective study, we assessed the FM NGS data of patients diagnosed with recurrent or metastatic HNSCC. These patients underwent NGS from January 1, 2019, through December 31, 2021, at Shanghai East Hospital. The study secured approval from the Shanghai East Hospital’s ethics committee (Approval No. 2023-027). Owing to the study’s retrospective design, the committee granted a waiver for individual patient consent.

Participants were selected based on the following inclusion criteria: 1) diagnosis of recurrent or metastatic HNSCC; 2) completion of FM NGS testing; 3) an Eastern Cooperative Oncology Group (ECOG) performance status (PS) ranging from 0 to 1; 4) satisfactory organ function. Patients were excluded if they: 1) had nasopharyngeal cancer; 2) presented with an incomplete medical history or lacked pertinent records; 3) were devoid of follow-up data.

### Next-generation sequencing

All cases underwent meticulous review to validate the diagnosis, ensuring no admixture of other lesion components. Specimens were preserved in 10% neutral buffered formalin. Subsequently, formalin-fixed paraffin-embedded (FFPE) tissue blocks were dispatched to Foundation Medicine (Cambridge, MA, USA) for FM NGS, following the methodology delineated in prior publications (14). The FM NGS panel deployed for this study encompassed 324 genes (6). From every specimen, a minimum of 50 ng of DNA was extracted. Using the HiSeq 4000 platform by Illumina, Inc. (San Diego, CA, USA), the libraries underwent sequencing to achieve a uniformly profound depth, targeting a median coverage surpassing 500×. Furthermore, it aimed to cover over 99% of the exons at a depth exceeding 100× (6, 14). This assay is adept at concurrently detecting base substitutions, deletions, insertions, gene fusions, and gene amplifications. Additionally, it facilitates the assessment of microsatellite instability and tumor mutational burden (TMB) (6).

### Data collection

Patient demographics (including age and sex), clinical presentations (such as primary HNSCC site and its recurrent/metastatic status), tumor attributes (incorporating HPV status, combined positive score [CPS], and TMB), and immunotherapy details were meticulously extracted from patient medical records. Overall survival (OS) was defined as the interval from the diagnosis of recurrent or metastatic HNSCC to death from any cause. For those who underwent immunotherapy, the OS was calculated from the onset of such treatment. Progression-free survival (PFS) was quantified as the duration from the diagnosis of recurrent or metastatic HNSCC to either disease progression or death from any cause, whichever occurred first. Follow-ups concluded at the last recorded patient visit. Overall response was characterized as a complete or partial response. Disease control encompassed scenarios of complete response, partial response, or stable disease.

### Pathway assignment

The 324 genes encompassed in the FM NGS panel were systematically aligned to the curated pathways as listed in the National Cancer Institute (NCI) Pathway Interaction Database (PID) (19). In instances where genes corresponded to multiple pathways within the NCI PID, preference was given to pathways with the most significant proportions of aligned genes.

### Statistical analysis

Patients were stratified based on the presence of a CDKN2A loss-of-function (LOF) mutation versus its wild-type (WT) counterpart. A focused subgroup analysis was undertaken for those who underwent immunotherapy. Categorical variables were articulated as n (%) and subjected to analysis via the chi-square test or Fisher’s exact test, as appropriate. To discern factors independently correlating with OS, a multivariable Cox regression analysis was employed. OS distributions were delineated using the Kaplan-Meier methodology, and the resulting survival curves underwent comparison through the log-rank test. All statistical computations were executed utilizing SPSS 23.0 (IBM, Armonk, NY, USA) and Prism 9 (GraphPad Software Inc., San Diego, CA, USA). A two-sided *P*-value below 0.05 was earmarked as the threshold for statistical significance.

## Results

### Baseline characteristics of the patients

**Figure 1:**
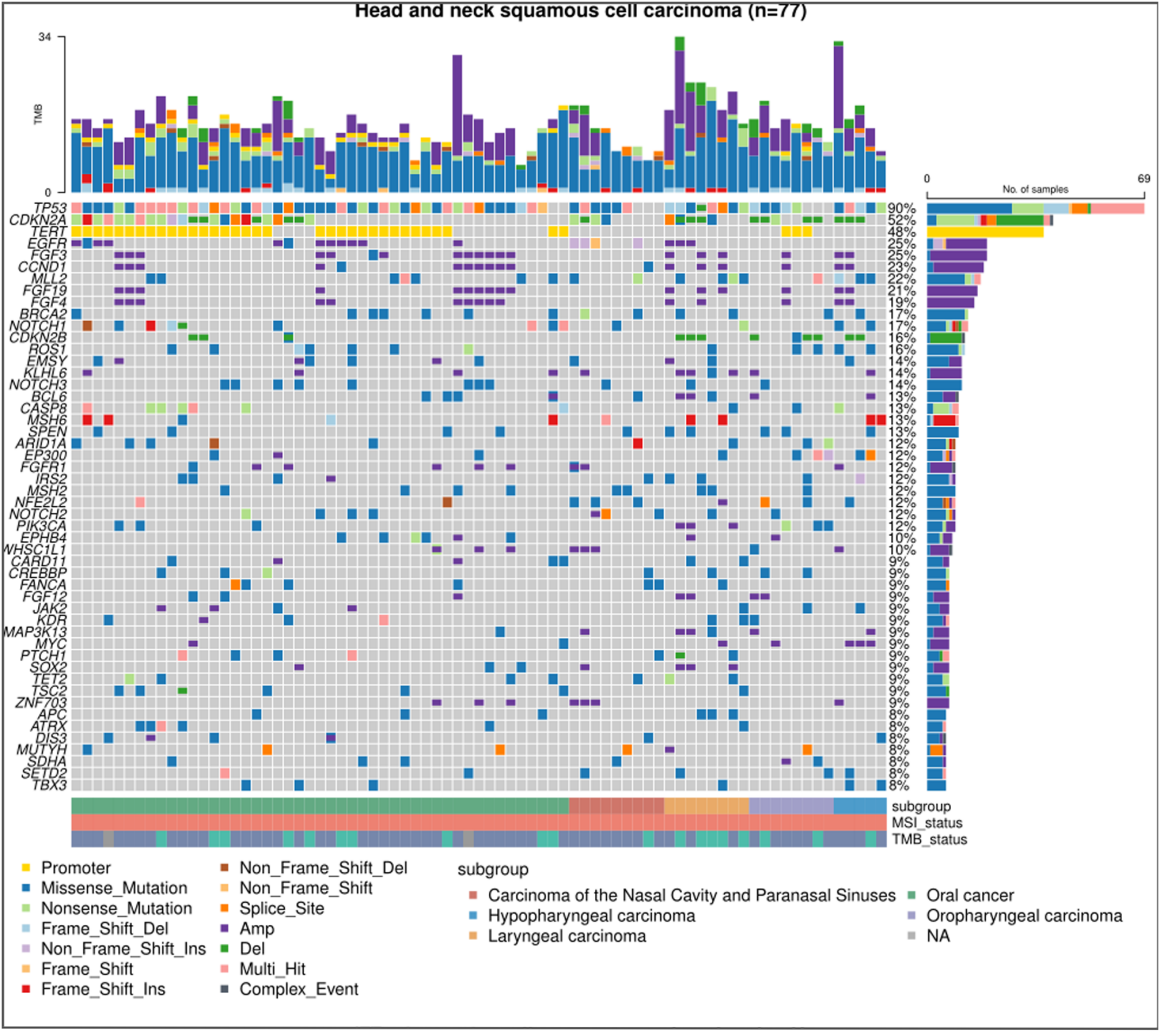
Mutational landscape in HNSCC via next-generation sequencing. The chart displays the most predominant gene mutations in the cohort of 77 patients diagnosed with recurrent or metastatic head and neck squamous cell carcinoma (HNSCC). Each column signifies an individual HNSCC case, while each row indicates the status of a specific gene: grey for wild-type and colored for mutated.

Of the 104 eligible patients, 77 were finally included. The median age was 56 years, spanning a range from 29 to 84 years. A predominant 80.5% were male, while 61.0% were diagnosed with oral HNSCC. The breakdown of disease recurrence and metastasis was as follows: 63.6% exhibited local recurrence, 12.9% had distant metastasis, and 23.4% displayed both local recurrence and distant metastasis. A significant 93.5% presented with a low TMB, 74.0% had undergone more than two lines of treatment, and 80.5% had been subjected to immunotherapy. Comparative analysis between the CKDN2A LOF and WT groups revealed no statistically significant disparities (all *P*>0.05) (**Table 1**). **Figure 1** delineates the most frequently observed gene mutations across the cohort of 77 patients.

**Table 1:** Baseline characteristics (all patients).

Variables	Number of patients (%)	CDKN2A LOF	CDKN2A WT	*P*
All	77	35	42	
Age, years, median (range)	56 (29-84)	55 (29-74)	59 (34-84)	
≥60 years old	29	9	20	0.0608
<60 years old	48	26	22	
Sex				
Male	62 (80.5)	31 (88.6)	31 (73.8)	0.1495
Female	15 (19.5)	4 (11.4)	11 (26.2)	
Primary site				
Oral cancer	47 (61.0)	19 (54.3)	28 (66.7)	0.7516
Oropharyngeal cancer	8 (10.4)	5 (14.3)	3 (7.1)	
Laryngeal cancer	8 (10.4)	4 (11.4)	4 (9.5)	
Hypopharynx cancer	5 (6.5)	3 (8.6)	2 (4.8)	
Nasal and sinus cancer	9 (11.7)	4 (11.4)	5 (11.9)	
Tumor site				
Local recurrence	49 (63.6)	21 (60.0)	28 (66.7)	0.6083
Distant metastasis	10 (12.9)	4 (11.4)	6 (14.3)	
Both	18 (23.4)	10 (28.6)	8 (19.0)	
HPV (oropharyngeal cancer)				
Positive	1 (1.3)	0	1 (2.4)	0.3750
Negative	7 (9.1)	5 (14.3)	2 (4.8)	
CPS				
<1	12 (15.6)	4 (11.4)	8 (19.0)	0.7612
1-19	20 (25.9)	9 (25.7)	11 (26.2)	
≥20	18 (23.4)	8 (22.9)	10 (23.8)	
NA	27 (35.1)	14 (40.0%)	13 (31.0)	
Tumor mutational burden				
High (≥10 mutations/Mb)	5 (6.5)	2 (5.7)	3 (7.1)	
Low (<10 mutations/Mb)	72 (93.5)	33 (94.3)	39 (92.9)	
Number of treatment lines				
Median (range)	3 (1-9)	3 (1-9)	3 (1-8)	0.6113
First line	20 (26.0)	8 (22.9)	12 (28.6)	
≥ two lines	57 (74.0)	27 (77.1)	30 (71.4)	
Receive immunotherapy				
Yes	62 (80.5)	27 (77.1)	35 (83.3)	0.5699
No	15 (19.5)	8 (22.9)	7 (16.7)	

LOF, loss-of-function; WT, wild-type; HPV, human papillomavirus; CPS, combined positive score; NA, not available.

### Prognosis

**Figure 2:**
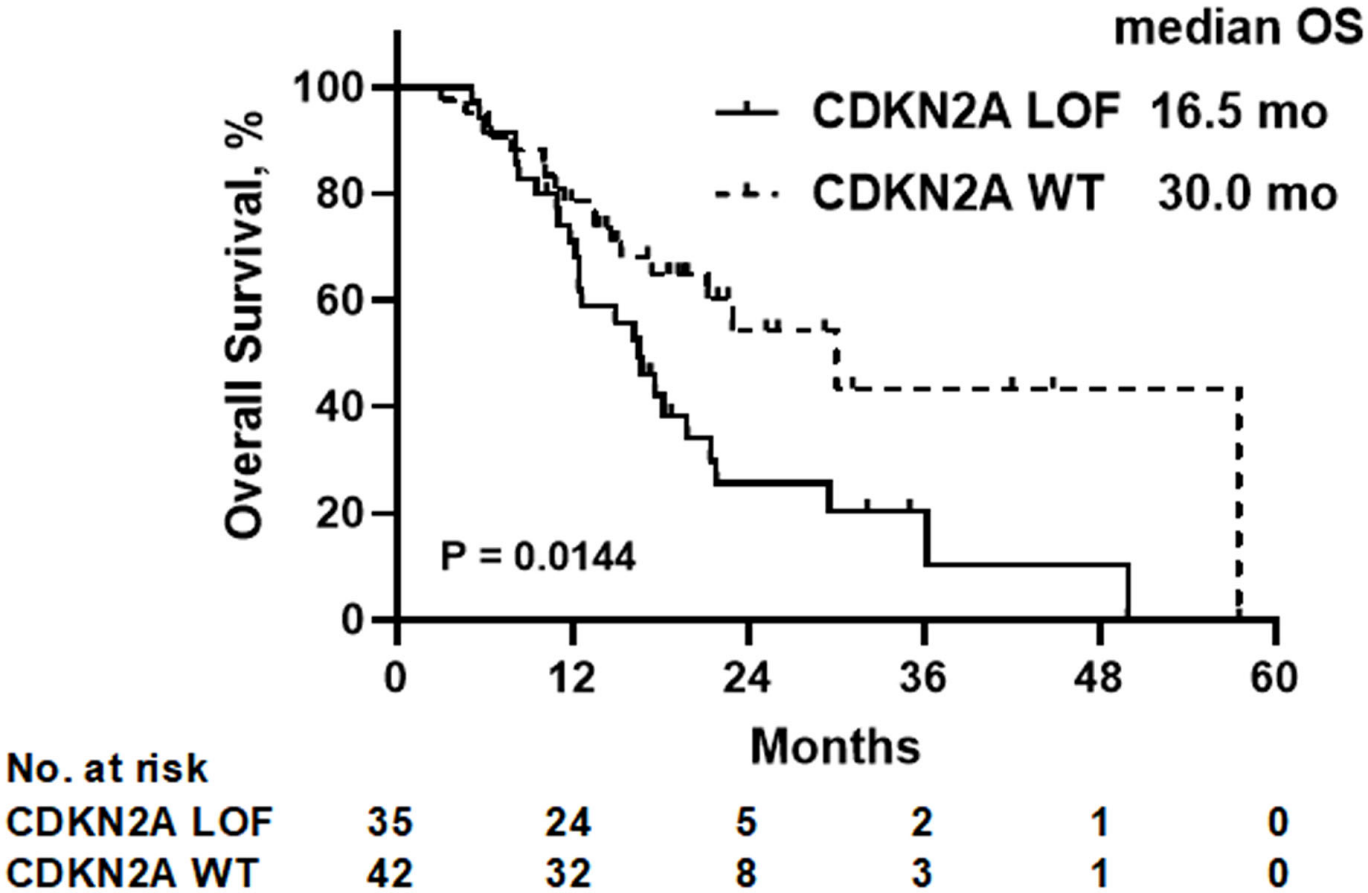
Overall survival (OS) of all patients according to the CDKN2A gene status. LOF, loss-of-function; WT, wild-type.

With a median follow-up duration of 22.6 months, the median OS was determined to be 16.5 months for the CDKN2A LOF group, contrasting with 30.0 months for the CDKN2A WT group (*P*=0.014) (**Figure 2**). As detailed in **Table 2**, both female gender (hazard ratio [HR]=4.526, 95% confidence interval [CI]: 1.934-10.180, *P*=0.0003) and presence of CDKN2A LOF (HR=2.311, 95%CI: 1.156-4.748, *P*=0.019) emerged as independent factors associating with increased mortality risk in patients diagnosed with recurrent or metastatic HNSCC.

**Table 2:** Univariate and multivariable analysis for overall survival (all patients).

Variables		Univariate analysis			Multivariate analysis		
		HR	95% CI	*P*	HR	95% CI	*P*
Sex	Male	1			1		
	Female	3.539	1.624 - 7.320	0.0009	4.526	1.934 - 10.18	0.0003
Age	≥60	1			1		
	<60	1.879	0.9796 - 3.849	0.0680	1.735	0.8321 - 3.847	0.1551
Primary site	Oral cancer	1			1		
	Oropharyngeal cancer	0.8634	0.2535 - 2.237	0.2716	0.5177	0.1416 - 1.504	0.2633
	Laryngeal cancer	0.6493	0.2136 - 1.610	0.8551	0.6374	0.1841 - 1.888	0.4405
	Hypopharynx cancer	1.304	0.3082 - 3.762	0.4302	0.8184	0.1709 - 2.890	0.7750
	Nasal and sinus cancer	1.351	0.4972 - 3.121	0.6538	1.058	0.3693 - 2.606	0.9081
Tumor site	Local recurrence	1			1		
	Distant metastasis	0.6940	0.2279 - 1.735	0.4715	0.5959	0.1702 - 1.719	0.3731
	Both	2.155	1.088 - 4.152	0.0234	1.795	0.8298 - 3.756	0.1266
Immunotherapy	Yes	1			1		
	No	0.6337	0.2175 - 1.475	0.3400	0.5312	0.1757 – 1.319	0.2090
CDKN2A	WT	1			1		
	LOF	2.114	1.154 - 3.973	0.0168	2.311	1.156 - 4.748	0.0194

HR, hazard ratio; CI, confidence interval; LOF, loss-of-function; WT, wild-type.

### Immunotherapy subgroup analysis

**Figure 3:**
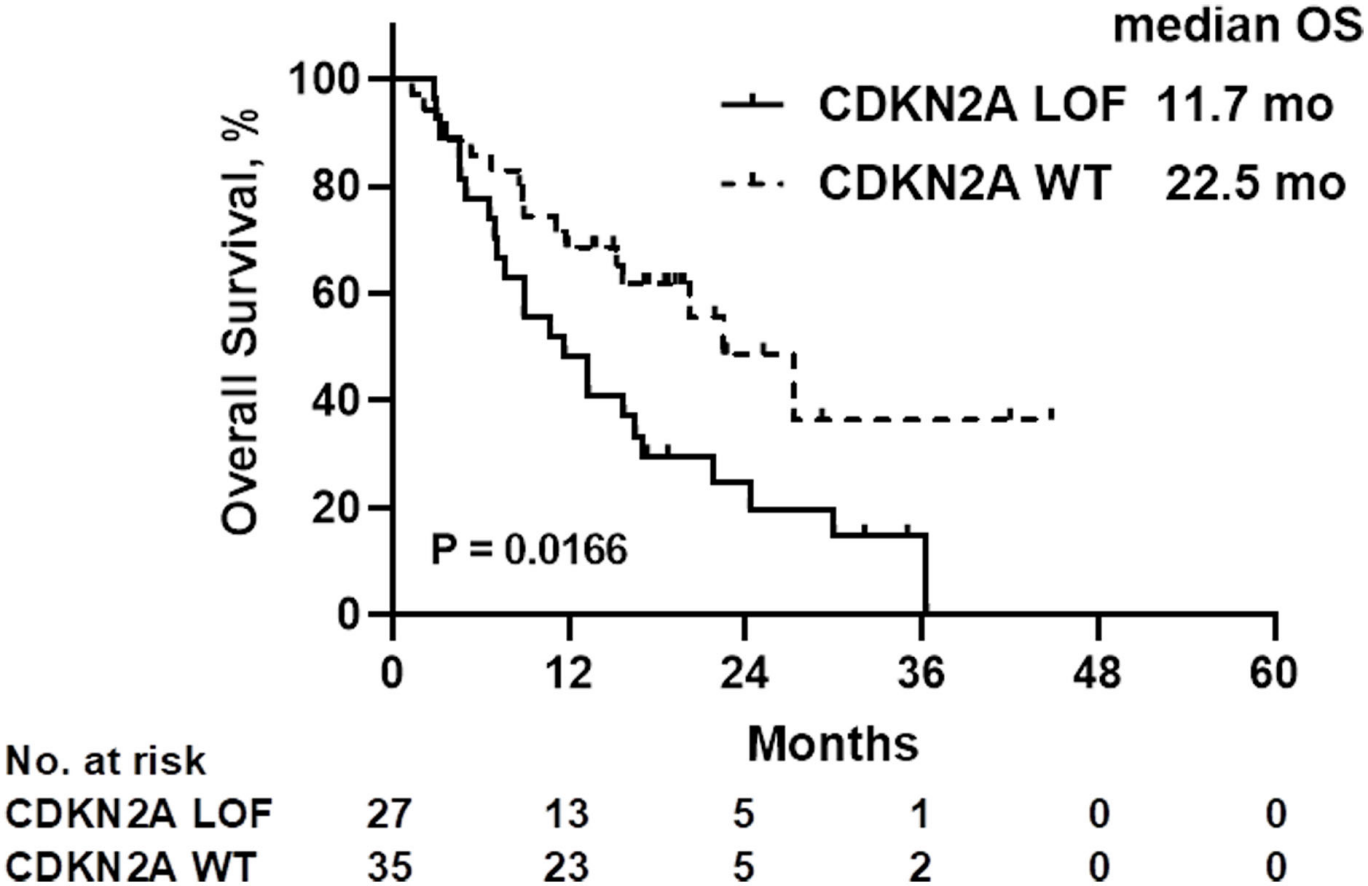
Overall survival (OS) of patients treated with immunotherapy, according to the CDKN2A gene status. LOF, loss-of-function; WT, wild-type.

A comparison of patient characteristics between the two groups, as detailed in **Table 3**, revealed no statistically significant differences (all *P*>0.05). Of those undergoing immunotherapy, the observed overall response rate was 33.9% (21/62), with a breakdown of 25.9% for the CDKN2A LOF group and 40.0% for the CDKN2A WT group (*P*=0.288). The disease control rate was 62.9% (39/62), bifurcating into 59.3% for the CDKN2A LOF group and 65.7% for the CDKN2A WT group (*P*=0.791). Both groups had a median PFS of 4.2 months (*P*=0.356). The median OS was 19.8 months, with 11.7 months for the CDKN2A LOF group and 22.5 months for the CDKN2A WT group (*P*=0.017) (**Figure 3**). Factors such as female gender (HR=4.022, 95%CI: 1.417-10.710, *P*=0.006), presence of CDKN2A LOF (HR=4.389, 95%CI: 1.782-11.460, *P*=0.002), and a CPS score below 1 (HR=17.20, 95%CI: 4.134-79.550, *P*<0.0001) emerged as independent associated factors of mortality in patients with recurrent or metastatic HNSCC undergoing immunotherapy (**Table 4**).

**Table 3:** Baseline characteristics (patients receiving immunotherapy).

Variables	Number of patients (%)	CDKN2A LOF	CDKN2A WT	*P*
Number of patients	62	27	35	
Age, median (range) (years old)	56 (34-74)	54 (38-74)	58 (34-70)	
≥60	23 (37.10)	7 (25.93)	16 (45.71)	0.1227
<60	39 (62.90)	20 (74.07)	19 (54.29)	
Sex				
Male	51 (82.26)	24 (88.89)	27 (77.14)	0.3209
Female	11 (17.74)	3 (11.11)	8 (22.86)	
Primary site				
Oral cancer	36 (58.06)	14 (51.85)	22 (62.86)	0.6058
Oropharyngeal cancer	8 (12.90)	5 (18.52)	3 (8.57)	
Laryngeal cancer	7 (11.29)	4 (14.81)	3 (8.57)	
Hypopharynx cancer	4 (6.45)	2 (7.41)	2 (5.71)	
Nasal and sinus cancer	7 (11.29)	2 (7.41)	5 (14.29)	
Tumor site				
Local recurrence	35 (56.45)	13 (48.15)	22 (62.86)	0.3248
Distant metastasis	10 (16.13)	4 (14.81)	6 (17.14)	
Both	17 (27.42)	10 (37.04)	7 (20.00)	
CPS				
<1	10 (16.13)	2 (7.41)	8 (22.86)	0.3312
1-19	15 (24.19)	7 (25.93)	8 (22.86)	
≥20	17 (27.42)	7 (25.93)	10 (28.57)	
NA	20 (32.26)	11 (40.74)	9 (25.71)	
Tumor mutational burden				
High (≥10 mutations/Mb)	5 (8.06)	2 (7.41)	3 (8.57)	>0.9999
Low (<10 mutations/Mb)	57 (91.94)	25 (92.59)	32 (91.43)	
Number of treatment lines				
First line	30 (48.39)	12 (44.44)	18 (51.43)	0.6175
≥ 2 lines	32 (51.61)	15 (55.56)	17 (48.57)	
Immunotherapy regimen				
Single-drug immunotherapy	28 (45.16)	13 (48.15)	15 (42.86)	0.7981
Combination immunotherapy	34 (54.84)	14 (51.85)	20 (57.14)	
PD-1 + chemotherapy	16 (25.81)	7 (25.93)	9 (25.71)	0.9663
PD-1 + targeted therapy	18 (29.03)	7 (25.93)	11 (31.43)	
PD-1 + anti-EGFR agents	12 (19.35)	5 (18.52)	7 (20.00)	
PD-1 + anti-angiogenic drugs	4 (6.45)	1 (3.70)	3 (8.57)	
PD-1 + others	2 (3.23)	1 (3.70)	1 (2.86)	

LOF, loss-of-function; WT, wild-type; HPV, human papillomavirus; CPS, combined positive score.

**Table 4:** Univariate and multivariable analysis for overall survival (patients receiving immunotherapy).

Variables		Univariate analysis			Multivariate analysis		
		HR	95% CI	*P*	HR	95% CI	*P*
Sex	Male	1			1		
	Female	2.643	1.085 - 5.864	0.0220	4.022	1.417 - 10.71	0.0063
Age	≥60	1			1		
	<60	2.262	1.133 - 4.844	0.0261	1.769	0.6641 - 4.951	0.2603
Primary site	Oral cancer	1			1		
	Oropharyngeal cancer	0.6160	0.1797 - 1.615	0.3743	0.3435	0.07028 - 1.312	0.1458
	Laryngeal cancer	0.5528	0.1587 - 1.477	0.2854	0.7897	0.1134 - 3.635	0.7838
	Hypopharynx cancer	2.652	0.6127 - 8.049	0.1240	2.462	0.4189 - 12.25	0.2845
	Nasal and sinus cancer	1.142	0.3328 - 3.005	0.8076	0.8036	0.1667 - 3.064	0.7648
Tumor site	Local recurrence	1			1		
	Distant metastasis	0.8655	0.3283 - 2.042	0.7531	0.3397	0.08219 - 1.174	0.1075
	Both	1.732	0.8127 - 3.584	0.1433	1.312	0.3997 - 4.117	0.6449
CDKN2A	WT	1			1		
	LOF	1.972	1.044 - 3.815	0.0383	4.389	1.782 - 11.46	0.0016
CPS	≥20	1			1		
	1-19	1.920	0.7136 - 5.386	0.1967	2.946	0.9512 - 9.615	0.0632
	<1	5.110	1.854 - 14.63	0.0016	17.20	4.134 - 79.55	< 0.0001
	NA	1.305	0.5156 - 3.553	0.5816	1.278	0.4066 - 4.152	0.6735
Tumor mutational burden	High	1			1		
	Low	0.5162	0.1223 - 1.471	0.2810	0.9461	0.1079 - 8.005	0.9588
Number of treatment lines	First line	1			1		
	≥2 lines	1.864	0.9784 - 3.658	0.0619	1.157	0.4217 - 3.266	0.7782
Immunotherapy model	Single drug	1			1		
	Combination	1.628	0.8481 - 3.248	0.1511	1.799	0.7666 - 4.282	0.1761

HR, hazard ratio; CI, confidence interval; LOF, loss-of-function; WT, wild-type; CPS, combined positive score.

### CDKN2A alterations

**Figure 4:**
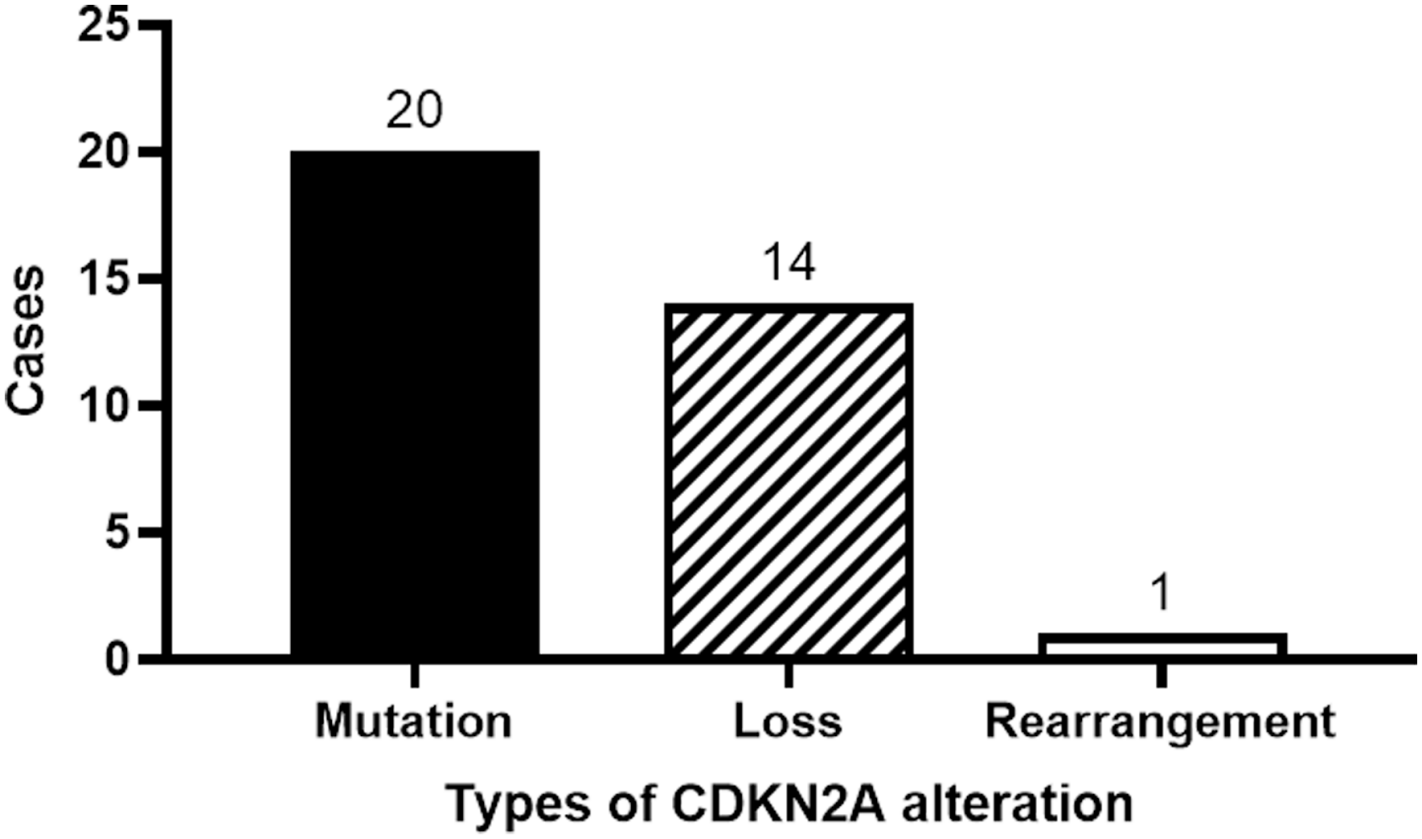
Classification of CDKN2A gene alterations.

**Figure 5:**
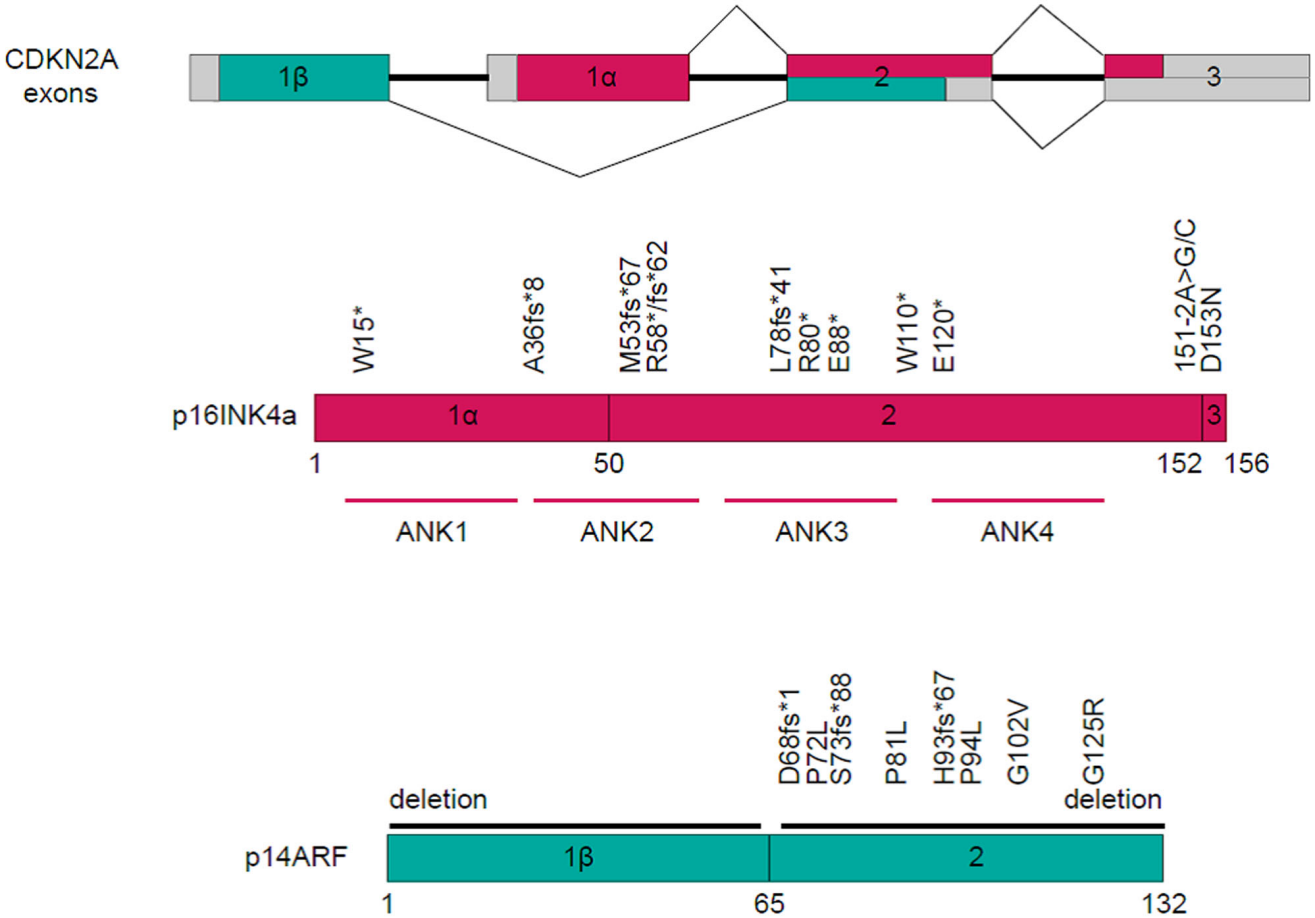
Schematic representation of mutation sites within the CDKN2A Gene.

Twenty patients manifested CDKN2A mutations, fourteen exhibited a reduction in the CDKN2A copy number, while one displayed gene rearrangement (**Figure 4**). A comprehensive depiction of these alterations can be found in **Figure 5**.

## Discussion

The role of CDKN2A in HNSCC, though studied, has not been exhaustively elucidated, especially in the context of immunotherapy. Our study aims to bridge this knowledge gap by examining the prognostic implications of CDKN2A alterations in patients with recurrent and metastatic HNSCC undergoing immunotherapy, shedding light on a relatively under-explored facet of HNSCC management. Our findings underscore that LOF alterations in the CDKN2A gene are intrinsically linked with diminished survival outcomes in HNSCC patients, encompassing those undergoing immunotherapy.

Historically, the CDKN2A gene emerges as the second most frequently mutated gene in HNSCC (20, 21). Between 57% and 67% of HNSCC patients exhibit CDKN2A mutations (22, 23). Notably, in our research, we observed a mutation rate of 45.5% for CDKN2A, marginally lower than previously reported metrics. Such divergence might be attributable to the unique patient demographics of our study. Among our cohort, mutations in CDKN2A were predominant, accounting for 57.1% (20 out of 35), followed by a reduction in copy number (40.0%, or 14 out of 35) and a singular instance of gene rearrangement (2.9%).

Germline mutations within CDKN2A have affiliations with hereditary cancer syndromes (24). On the somatic front, mutations in CDKN2A correlate with poorer prognostic outcomes in HNSCC patients (20, 21). The inactivation of CDKN2A via hypermethylation processes is implicated in the carcinogenesis, progression, and metastatic patterns of HNSCC (17). A reduced CDKN2A copy number stands as a harbinger of adverse prognosis (16), while its mutation often coincides with a heightened TMB, another indicator of poor prognosis in HNSCC (25). Previous studies have concretized the association between CDKN2A LOF and diminished prognosis. For instance, Chen et al. (16) identified a direct association between the loss of CDKN2A copy number and heightened mortality in NHSCC patients, irrespective of chemotherapy or radiotherapy interventions. Analogous findings have surfaced in the context of patients subjected to chemoradiotherapy for advanced oro- and hypo-pharyngeal HNSCC (26) as well as in both recurrent and non-recurrent oral HNSCC subsets (27, 28). Reinforcing these narratives, our present investigation discerned no significant variance in treatment modalities between CDKN2A LOF and WT groups. Nonetheless, CDKN2A LOF was determined to be an independent predictor of a truncated OS, echoing the insights offered by the aforementioned literature.

Immunotherapy represents an innovative treatment approach in HNSCC, consistently demonstrating appreciable survival advantages (6, 29–31). The role of CDKN2A in immunotherapeutic responses is multifaceted. For instance, its association with resistance to immunotherapy has been noted in urothelial carcinoma (32). In the context of melanoma, deletions in CDKN2A appear to attenuate the efficacy of immune checkpoint inhibitors (33, 34). When shifting the lens to non-small-cell lung cancer (NSCLC), a homozygous loss of CDKN2A correlates with diminished PD-L1 expression (35). Strikingly, even amidst high levels of PD-L1 expression and an elevated TMB, CDKN2A LOF was linked to suboptimal outcomes following immunotherapy in NSCLC patients (36). Furthermore, both pancreatic tumors and gliomas exhibiting CDKN2A LOF demonstrated a decreased cytolytic activity (37, 38).

Echoing these findings, our current study revealed a markedly abbreviated survival span in HNSCC patients treated with immunotherapy within the CDKN2A LOF group, in contrast to their WT counterparts. This observation might be attributable to the altered tumor microenvironment characteristic of CDKN2A LOF tumors (39, 40). Notably, our dataset had a limited subset of patients who did not undergo immunotherapy, making comparative analyses challenging. Besides, Liu et al. (41) reported a correlation between CDKN2A mutations and subpar responses to immune checkpoint inhibitors, particularly in patients with resectable HNSCC undergoing neoadjuvant immunotherapy. Nonetheless, the intrinsic relationship between CDKN2A LOF and poorer prognostic outcomes in immunotherapy recipients implies potential ramifications in patient selection for immunotherapy, a treatment modality that, despite its merits, is costly and not devoid of associated risks. Such a hypothesis warrants exploration in subsequent research endeavors.

Among patients undergoing immunotherapy, our findings reveal that a CPS of less than 1 is intrinsically tied to an inferior prognostic trajectory compared to those boasting a CPS greater than 20. This aligns with the diminished OS observed in patients with a CPS below 1 in the KEYNOTE-048 trial (42). While our data intriguingly suggest a gender-based association with OS, the current dataset lacks the granularity to elucidate this relationship. Factors such as the limited sample size and the gender disparity might introduce potential confounders in the statistical interpretations.

This study is not without its constraints. Being a retrospective study anchored to chart-recorded data inherently restricts the depth of clinical and pathological evaluations surrounding CDKN2A LOF. The study’s monocentric nature confined the sample size. Given the limited cohort and the infrequent manifestation of other mutated genes, it became infeasible to probe the interplay among various mutated genes. The diminutive subset of patients without immunotherapy further precluded a comparative evaluation between those receiving and not receiving immunotherapy within the CDKN2A LOF and WT categories. To truly unpack the prognostic implications of CDKN2A LOF, especially its interrelation with immunotherapy outcomes in HNSCC patients, there’s a pressing need for more extensive studies.

In conclusion, our study underscores that CDKN2A LOF stands as an independent harbinger of adverse prognosis in patients grappling with recurrent metastatic HNSCC, inclusive of those on immunotherapy.

## Data availability statement

The data presented in this study are available in article or supplementary material.

## Ethics statement

The studies involving humans were approved by the Ethics Committee of Shanghai East Hospital. The studies were conducted in accordance with the local legislation and institutional requirements. The ethics committee/institutional review board waived the requirement of written informed consent for participation from the participants or the participants’ legal guardians/next of kin due to the retrospective nature of the study.

## Author contributions

LX: Data curation, Formal Analysis, Project administration, Writing – original draft, Writing – review & editing. WT: Data curation, Writing – review & editing. JZ: Data curation, Writing – review & editing. JX: Writing – review & editing. QL: Writing – review & editing. XG: Writing – review & editing. FL: Writing – review & editing. WZ: Writing – review & editing. YG: Conceptualization, Data curation, Formal Analysis, Investigation, Methodology, Project administration, Software, Supervision, Writing – original draft, Writing – review & editing.
